# Timing, causes, predictors and prognosis of switching from peritoneal dialysis to hemodialysis: a prospective study

**DOI:** 10.1186/1471-2369-10-3

**Published:** 2009-02-06

**Authors:** Bernard G Jaar, Laura C Plantinga, Deidra C Crews, Nancy E Fink, Nasser Hebah, Josef Coresh, Alan S Kliger, Neil R Powe

**Affiliations:** 1 Department of Medicine, Johns Hopkins University School of Medicine, Baltimore, MD, USA; 2 Department of Epidemiology, Johns Hopkins Bloomberg School of Public Health, Baltimore, MD, USA; 3 Nephrology Center of Maryland, Baltimore, Maryland, USA; 4 Dialysis Clinic, Inc., Nashville, TN, USA; 5 Department of Medicine, Hospital of St. Raphael, New Haven, CT, USA; 6 Department of Health Policy and Management, Johns Hopkins Bloomberg School of Public Health, Baltimore, MD, USA

## Abstract

**Background:**

The use of peritoneal dialysis (PD) has declined in the United States over the past decade and technique failure is also reportedly higher in PD compared to hemodialysis (HD), but there are little data in the United States addressing the factors and outcomes associated with switching modalities from PD to HD.

**Methods:**

In a prospective cohort study of 262 PD patients enrolled from 28 peritoneal dialysis clinics in 13 U.S. states, we examined potential predictors of switching from PD to HD (including demographics, clinical factors, and laboratory values) and the association of switching with mortality. Cox proportional hazards regression was used to assess relative hazards (RH) of switching and of mortality in PD patients who switched to HD.

**Results:**

Among 262 PD patients, 24.8% switched to HD; with more than 70% switching within the first 2 years. Infectious peritonitis was the leading cause of switching. Patients of black race and with higher body mass index were significantly more likely to switch from PD to HD, RH (95% CI) of 5.01 (1.15–21.8) for black versus white and 1.09 (1.03–1.16) per 1 kg/m^2 ^increase in BMI, respectively. There was no difference in survival between switchers and non-switchers, RH (95% CI) of 0.89 (0.41–1.93).

**Conclusion:**

Switching from PD to HD occurs early and the rate is high, threatening long-term viability of PD programs. Several patient characteristics were associated with the risk of switching. However, there was no survival difference between switchers and non-switchers, reassuring providers and patients that PD technique failure is not necessarily associated with poor prognosis.

## Background

Hemodialysis (HD) and peritoneal dialysis (PD) represent the two main modalities for renal replacement therapy. PD is typically considered a home dialysis program, as the patients have the autonomy to perform the treatment in their home environment, whereas most HD patients must travel to a dialysis center, usually three times a week, to receive their treatment. Despite the potential benefit of PD compared to HD in quality of life [1] and associated patient satisfaction [2], prevalent use of PD has declined in the United States since 1994–1995, by as much as 67% in some regions of the country [3]. The number of incident end-stage renal disease (ESRD) patients initiating PD has also declined over the same time period [3]. This decline in PD utilization has been observed not only in the United States but also in Europe and elsewhere [3,4]. Technique failure is known to be much higher in PD than HD patients [5-7] and this likely plays a significant role in the declining prevalence of PD utilization. Peritonitis has been described as one of the leading causes of transfer from PD to HD [7-9] and only a small group of patients can return to PD after severe peritonitis and Tenckhoff catheter removal [10].

Over the past decade, very few studies in the United Sates have analyzed both the cause of switching from PD to HD and the timing of this switching process after initiation of PD. Further, there is a paucity of studies, particularly in the United States, aimed at identifying risk factors associated with switching from PD to HD in ESRD patients and subsequent patient outcomes. The purpose of this study was to determine patient characteristics associated with the risk of switching from PD to HD and to assess patient survival following dialysis modality switches in a cohort of incident peritoneal dialysis patients.

## Methods

### Study design and research population

The Choices for Healthy Outcomes in Caring for ESRD (CHOICE) is a national prospective cohort study of incident dialysis patients [11]. For the purposes of this study, we limited our sample to 262 white and black peritoneal dialysis patients from the CHOICE cohort. From October 1995 to June 1998, participants from 13 states were enrolled at 28 clinics offering peritoneal dialysis and associated with Dialysis Clinic, Inc. (Nashville, TN; *n *= 178), New Haven CAPD (New Haven, CT; *n *= 82) or St. Raphael's Hospital (New Haven, CT; *n *= 2). Eligibility criteria for enrollment included ability to provide informed consent for participation, age older than 17 years, and ability to speak English or Spanish. Median time from start of peritoneal dialysis to enrollment was 29 days, with 99% enrolling within 4 months of initiating dialysis. All participants gave written informed consent after Institutional Review Boards for Johns Hopkins University and clinical centers approved the study protocol.

### Data collection

#### Dialysis modality and switching

Dialysis modality was defined as the modality in use at 4 weeks after enrollment in the study (an average of 10 weeks after starting dialysis). This information was obtained from clinic records. The initial dialysis modality information was abstracted from the Centers for Medicare and Medicaid Services (CMS) ESRD Medical Evidence Report (Form 2728). All forms of PD (continuous ambulatory PD, continuous cycling PD and intermittent cycling PD) were combined as a single category. Patients were considered to have switched to hemodialysis (HD) when they changed from PD to HD and remained on the latter modality for at least 30 days. Causes of switch from PD to HD were ascertained from comprehensive chart review. Patients were censored for time to switch at transplantation, loss to follow-up, death, or last date of follow-up (December 31, 2004).

#### Demographic and clinical data

All patients completed a baseline self-report questionnaire and provided information on demographics, health behaviors, work history, medical history, and distance to dialysis unit. Late referral was defined as <4 months between first nephrologist evaluation and start of dialysis, as described previously [12]. Residual urine output, obtained from the patient baseline self-report questionnaire, was defined as the ability to make at least 250 cc (1 cup) of urine per day. Body mass index (BMI) was calculated using the standard formula weight (in kg)/[height (in meters)]^2^, based on the height and weight reported on the 2728 form. Comorbidity, referring to medical conditions other than the primary disease itself and the severity of those conditions, was assessed using the ICED, a medical record-derived index that has been demonstrated to predict death in dialysis populations [13,14]. ICED scores range from 0 to 3, with 3 as the highest severity level. It is a measure of both the presence and severity of comorbid conditions, as described previously. Baseline data for routine patient care were available for the following laboratory values: serum albumin, hemoglobin, total cholesterol, and serum creatinine. High-sensitivity C-reactive protein (CRP) level was assessed at a median of 5.0 months from dialysis initiation, using a colorimetric competitive enzyme-linked immunosorbent assay (coefficient of variation, 8.9%). Glomerular filtration rate (GFR) before dialysis initiation was estimated by the six-variable Modification of Diet in Renal Disease (MDRD) equation using serum creatinine obtained from the CMS Form 2728 [15].

#### Mortality ascertainment

Mortality information was ascertained from clinic report, medical records, National Death Index and CMS (death notification forms and Social Security records). Follow-up for mortality continued until death (*n *= 88), transplantation (*n *= 69), loss to follow-up [when patients left the study or study clinic (*n *= 97)], or the last follow-up date of December 31, 2004 (*n *= 8). Patients were followed for mortality for up to 8.9 years (average follow-up, 2 years). In sensitivity analyses, we also assessed mortality without loss to follow-up, by including deaths tracked by passive follow-up through death certificates of patients who left the study or the study clinic.

### Statistical analysis

We compared characteristics of patients who switched (switchers) to hemodialysis with those patients who remained on PD (non-switchers) by using *t *tests for continuous variables and Pearson's χ^2 ^tests for categorical variables. CRP was log-transformed to reduce skewness of distribution.

We used time-dependent Cox proportional hazards models to assess the risk factors for switching by analyzing the time to first switch from peritoneal dialysis to hemodialysis. Time-dependent analyses were performed to reduce lead-time bias, since, by definition, those who switched modality had to survive at least until the switch. In these analyses, all patients started as non-switchers, and if the patient switched the patient then became a switcher in the analyses. In multivariable models, we adjusted for potential confounders, including variables associated with both baseline modality and switching. We also used Cox proportional hazards models to assess the mortality risk of patients on peritoneal dialysis who switched to hemodialysis versus patients who remained on peritoneal dialysis, independent of differences in demographics (e.g., age, race, and employment status), clinical factors (e.g., ICED comorbidity score, diabetes mellitus status, history of cardiovascular disease, body mass index, and baseline residual urine output), and laboratory values (e.g., serum albumin and creatinine).

We also examined whether the mortality risk was similar by year of follow-up and among persons with different clinical characteristics by performing Cox proportional hazards analyses in subpopulations based on survival time, diabetes mellitus status, history of cardiovascular disease, baseline residual urine output and baseline serum albumin (< 3.5 g/dl versus ≥ 3.5 g/dl). We formally tested for interactions that had been found to be significant in previous studies by including interaction terms and testing their statistical significance in the full population models. Furthermore, we tested for and found no deviations from the proportional hazards assumption by examining the global test of Schoenfeld residuals, both overall and within each follow-up year. Finally, we accounted for possible dependence of observations within clinics [16] by performing fixed-effects modeling clustered on the dialysis clinic. Statistical analyses were performed using Stata version 8.2 (StataCorp, College Station, TX).

## Results

### Patient characteristics

Among the 262 peritoneal dialysis patients, 24.8% switched to hemodialysis during the study period. PD patients who switched to HD had higher BMI and serum creatinine at baseline and were less likely to be white and to have residual urine output at both baseline and 1 year of follow-up. There were no other demographic, clinical or laboratory differences between PD switchers and non-switchers at baseline (Table 1).

**Table 1:** Patient characteristics by peritoneal dialysis switching status

Characteristic	*N*	Non-switchers	Switchers	P
Total	262	197 (75.2%)	65 (24.8%)	--

**Demographic**				

Mean age at enrollment, years	262	54.7 ± 15.4	52.0 ± 13.0	0.202

Sex (% female)	262	42.1	47.7	0.433

Race (% white)	262	83.8	72.3	0.042

Education (% high school graduate)	225	84.2	75.4	0.131

Employment (% working)	262	28.4	20.0	0.181

Marital Status (% married)	238	68.9	62.3	0.341

Distance from clinic (% >30 miles)	216	28.3	28.1	0.973

**Clinical**				

Smoking status (% ever smoker)	227	61.5	60.7	0.914

Modality at start, from 2728 (% HD)	256	7.8	9.4	0.693

ICED score (%)	262			0.848

≤ 1		50.3	46.2	

2		25.4	27.7	

3		24.4	26.2	

Diabetes (% diabetic)	262	49.2	58.5	0.197

History of CVD (% positive)	262	52.3	41.5	0.133

History of CHD (% positive)	262	41.1	32.3	0.207

History of CHF (% positive)	262	40.6	29.2	0.101

Primary cause of renal failure (%)	259			0.341

Diabetes mellitus		44.8	53.9	

Hypertension		10.8	6.2	

Glomerulonephritis		44.3	40.0	

Late referral (% <4 months)	198	21.7	16.4	0.404

BMI, kg/m^2^	245	25.9 ± 5.6	28.2 ± 6.0	0.006

Residual urine output (%)	186			0.018

Not at baseline or follow-up at 1 year		14.6	19.6	

At baseline but not at follow-up at 1 year		17.7	33.9	

At baseline and at follow-up at 1 year		67.7	46.4	

**Laboratory**				

Mean baseline albumin, g/dl	248	3.57 ± 0.44	3.64 ± 0.38	0.316

Mean baseline hemoglobin, g/dl	246	11.3 ± 1.5	11.3 ± 1.5	0.766

Median CRP (IQR), μg/dl	162	2.6 (1.1–6.2)	3.5 (1.6–5.7)	0.638

Baseline creatinine, g/dl	252	7.08 ± 2.66	7.94 ± 2.63	0.029

Baseline cholesterol, mg/dl	220	207 ± 53	210 ± 57	0.746

Baseline GFR, cc/minute/1.73 m^2^	254	10.5 ± 0.26	10.3 ± 0.45	0.721

*By t-test (continuous variables) or χ^2 ^test (categorical variables)

### Causes of switching and time to switch from peritoneal dialysis to hemodialysis

**Figure 1:**
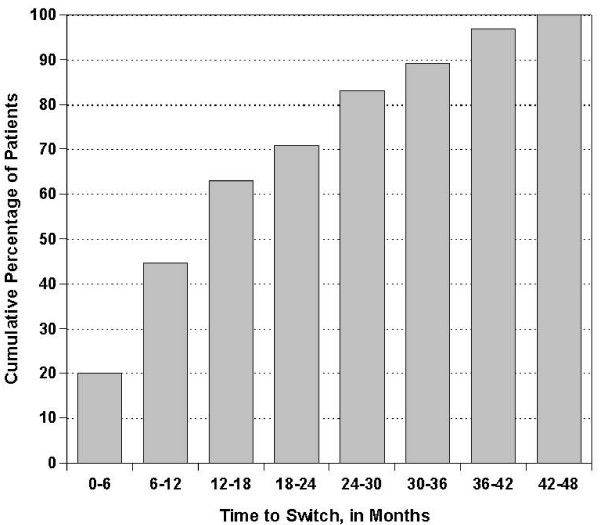
**Cumulative percentage of peritoneal dialysis patients by time from first dialysis to first switch to hemodialysis (switchers only)**.

**Figure 2:**
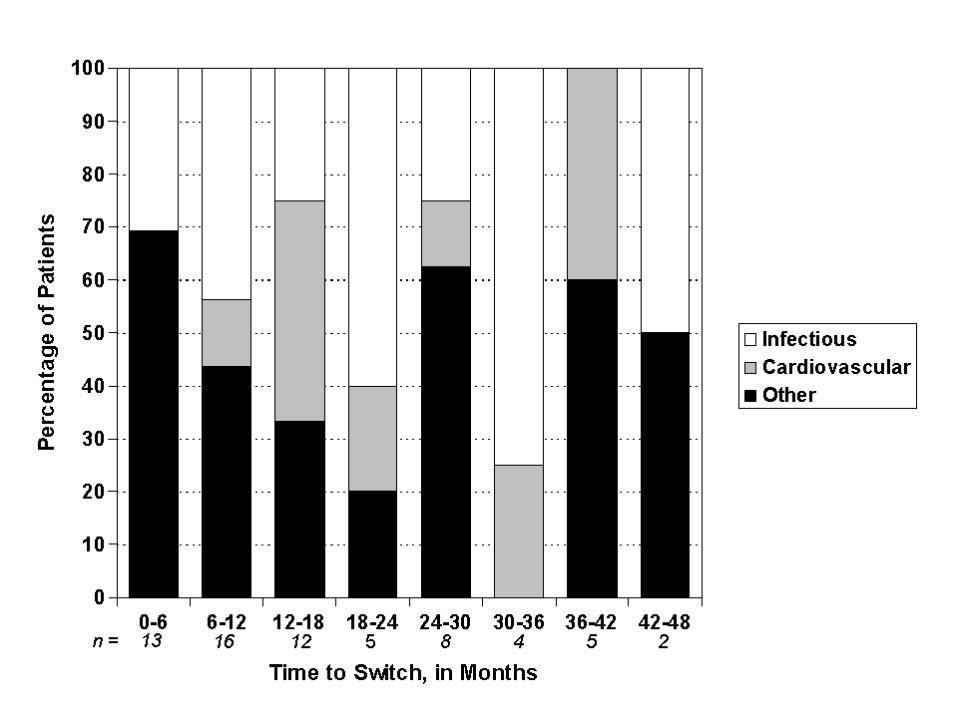
**Percentage of switching peritoneal dialysis patients by main causes of switch and by time from first peritoneal dialysis to hemodialysis switch**.

More than 40% of the PD patients who switched to HD did so within the first year after starting PD and more than 70% within the first two years (Figure 1). Infections (peritonitis and catheter-related) (36.9%) were the leading cause of switching from PD to HD, followed by cardiovascular (fluid overload) causes (18.5%). Less common causes of switching from PD to HD included abdominal surgery, pancreatitis/malnutrition, decreased mental capacity and abdominal wall defect (Table 2). Infectious peritonitis was a leading cause of switch from PD to HD during most of the follow-up period, whereas cardiovascular/fluid overload as a cause of switch became more dominant after the first year on peritoneal dialysis (Figure 2). Patients who switched due to infectious peritonitis versus any other cause were younger (47.1 versus 54.7 years, *P *= 0.02) and less likely to be white (52.2% versus 83.3%, *P *= 0.007); otherwise there were no other significant differences between these patients.

**Table 2:** Causes of switching from peritoneal dialysis to hemodialysis

Causes of Switching	Number (%) N = 65
Infection (peritonitis and catheter-related)	24 (36.9%)

Cardiovascular (fluid overload)	12 (18.5%)

Abdominal surgery	8 (12.3%)

Pancreatitis/malnutrition	7 (10.8%)

Decreased mental capacity	2 (3.1%)

Abdominal wall defect	1 (1.6%)

Unknown	11 (16.9%)

### Risk factors associated with switching from peritoneal dialysis to hemodialysis

In the unadjusted model, PD patients of black race were nearly 3 times more likely than white PD patients to switch from PD to HD; this risk became stronger after adjustment (Table 3). Patients who were less educated were 2.5 times more likely to switch from PD to HD, compared to patients who had at least a high school education; however, this association was not statistically significant. Patients living 30 miles or more from the dialysis clinic were 58% less likely to switch from PD to HD compared to patients living fewer than 30 miles from the dialysis clinic; but this association was marginally statistically significant. In both unadjusted and adjusted analyses, the risk of switching from PD to HD significantly increased by about 10% for each 1 kg/m^2 ^higher BMI. In the unadjusted analysis, for each 1 mg/dl higher serum baseline creatinine, there was a 13% increased risk of switching from PD to HD; however, this relationship was no longer statistically significant after adjustment. We found no significant risk of switching from PD to HD by age, employment status, or diabetes mellitus status (Table 3). Similarly, in sensitivity analyses, Index of Coexistent Disease (ICED) and residual urine output were not associated with the risk of switching from PD to HD (data not shown).

**Table 3:** Predictors of dialysis modality switching: relative hazards for switching versus non-switching (time to first switch) from peritoneal dialysis to hemodialysis

	Relative Hazards (95% CI)	
**Predictors**	**Unadjusted**	Adjusted*

Age (per 1-year increase)	1.00 (0.98–1.01)	0.98 (0.95–1.01)

Race		

White	1.00 (ref.)	1.00 (ref.)

Black	2.79 (1.25–6.23)	5.01 (1.15–21.8)

Education		

High school graduate or higher	1.00 (ref.)	1.00 (ref.)

Less than high school graduate	1.64 (0.83–3.23)	2.53 (0.98–6.55)

Employment		

Employed	1.00 (ref.)	1.00 (ref.)

Not employed	1.51 (0.76–3.01)	1.81 (0.66–4.94)

Distance to dialysis clinic		

Living less than 30 miles from clinic	1.00 (ref.)	1.00 (ref.)

Living 30 miles or more from clinic	0.65 (0.32–1.30)	0.42 (0.17–1.02)

Diabetes		

Nondiabetic	1.00 (ref.)	1.00 (ref.)

Diabetic	1.22 (0.71–2.12)	1.79 (0.74–4.33)

BMI (per 1 kg/m^2 ^increase)	1.10 (1.04–1.15)	1.09 (1.03–1.16)

Baseline creatinine (per 1 mg/dl increase)	1.13 (1.02–1.26)	1.13 (0.97–1.33)

Number (%) of patients switching/total number of patients by subpopulation: white, 47/212 (22.2%); black, 18/50 (36.0%); employed, 13/69 (18.8%); not employed, 52/193 (26.9%); high school graduate, 46/184 (25.0%); not high school graduate, 15/41 (36.6%); nondiabetic, 27/127 (21.3%); diabetic, 38/135 (28.2%); living at least 30 miles from dialysis clinic 41/155 (26.5%); living less than 30 miles from dialysis clinic 16/61 (26.2%).

* Adjusted model (n = 195) included age, race, education, employment, distance to dialysis clinic, diabetes mellitus status, BMI, baseline serum creatinine.

### Mortality risk associated with switching from peritoneal dialysis to hemodialysis

**Figure 3:**
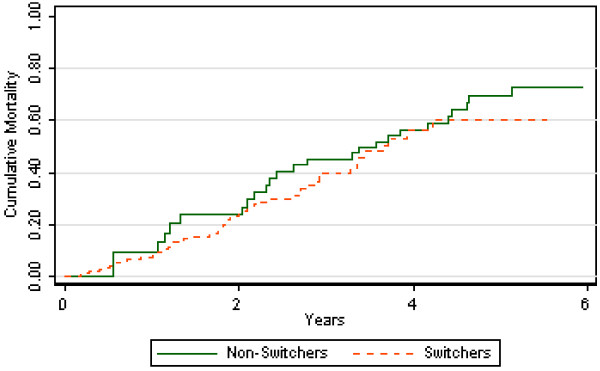
**Kaplan-Meier curve for mortality, peritoneal dialysis switchers versus non-switchers (P = 0.528 by log-rank)**.

The mortality rate per 100 patient-years was 18.5 for PD non-switchers versus 13.5 for PD patients who switched to HD (Table 4). The cumulative mortality did not differ between switchers and non-switchers (Figure 3). In the unadjusted analyses, switchers had a 6% decreased risk of death but the association was not statistically significant. After adjustment for demographics, clinical factors, and laboratory values, there was an 11% decreased risk of death in PD switchers compared to non-switchers but the results were not statistically significant (Table 4).

**Table 4:** Risk of mortality associated with switching from peritoneal dialysis to hemodialysis: relative hazards for mortality for switchers vs. non-switchers

	Relative Hazard (95% CI)	
	
Model	Non-switchers	Switchers
No. of deaths/total no. of patients	62/197	26/65

Incidence rate, per 100 patient-years	18.5	13.5


Unadjusted	1.00 (ref.)	0.94 (0.51–1.73)

Adjusted		

Model 1 (Demographics)	1.00 (ref.)	0.87 (0.46–1.66)

Model 2 (Model 1 + Clinical)	1.00 (ref.)	0.68 (0.33–1.40)

Model 3 (Model 2 + Laboratory)	1.00 (ref.)	0.89 (0.41–1.93)

In these analyses, all patients start as non-switchers; if the patient switched modality the patient then became a switcher on the date of switch.

*Demographics: age, race, employment; clinical: ICED, diabetes, CVD, BMI, residual urine output; laboratory: baseline albumin and creatinine. Fully adjusted models included all these variables.

In our sensitivity analyses, we found no significant decreased risk of death for PD switchers versus non-switchers by year of follow-up (1^st ^and 2^nd ^years) or after stratification by diabetes mellitus status, history of cardiovascular disease, baseline residual urine output, and baseline serum albumin (< 3.5 g/dl versus ≥ 3.5 g/dl) (data not shown). Additionally, when we examined the effects of including passive follow-up in our mortality data, results were similar and non-statistically significant (data not shown).

## Discussion

This prospective cohort study of incident PD patients showed that about 25% of patients switched to HD over time, with more than 70% of the switching occurring within the first 2 years of treatment. Peritonitis was the leading cause of this modality change. In this U.S. prospective cohort study, the leading independent predictors of dialysis modality switching from PD to HD were black race and higher BMI. Importantly, there was no statistically significant survival difference between PD patients who switched to HD compared to those who remained on PD.

At the end of 2005, only about 7.6% of U.S. dialysis patients were treated with PD, and this prevalence has been declining since the mid-1990s [3]. One of the factors certainly contributing to this low PD prevalence remains the unacceptable high transfer rate from PD to HD described in several cohorts [7,17-22]. In an older Italian study with long follow-up, 18% of PD patients switched modality, as compared to 2.8% of the HD patients [7]. In our U.S. incident cohort, this switching rate was 25% for PD patients switching to HD, compared to 5% for HD patients switching to PD [18]. In the Netherlands Cooperative Study on the Adequacy of Dialysis, 3-year technique survival was only 53% [21]. In a more recent U.S. cohort, Guo et al. [17] showed a significant trend towards decreasing transfer rates to HD during the first year on PD, from 19.6% in 1999 to 17.2% in 2001.

In agreement with previous studies, we found that infections remain the leading cause of switching from PD to HD, followed by cardiovascular causes, mainly fluid overload [9,17,23]. Infectious causes, which are generally preventable, were responsible for 28% of the transfers from PD to HD in a recent study by Mujais et al. [24]. However, in the early 1990s, this cause of dialysis modality transfer from PD to HD was reported to be as high as 49% [25]. Over the past few years, the use of the twin-bag and Y-set systems has certainly helped to decrease the peritonitis rate [26]. Ultrafiltration failure, leading to fluid overload, which was the next most important cause of transfer from PD to HD in our study, has been shown to increase with time on PD [23,27]; however, in our cohort, this trend was observed only during the first 18 months. We did not find an increasing number of ultrafiltration failures in our PD patients, possibly because a much smaller number of patients switched from PD to HD after 2 years. This ultrafiltration failure is a consequence of morphological and functional changes of the peritoneal membrane, including increased small solute transport and lymphatic absorption, over time [28,29]. Loss of residual renal function with decreasing urine output observed over time in this cohort is also another likely mechanism leading to more fluid overload as a cause of transfer from PD to HD.

We identified several patient characteristics associated with a higher risk of switching from PD to HD over time. Patients of black race were 5 times more likely than white patients to switch from PD to HD. This finding is in accordance with an older single-center study, which reported a significantly higher technique failure rate in black patients (39%) compared to white patients (8%) [30]. Patients with diabetes mellitus have also been reported to have a higher transfer rate from PD to HD in some cohorts [17,24] but certainly not all [31,32]. Similar to Huisman et al. [31] and Viglino et al. [32], we found no significant association between diabetes mellitus and modality transfer from PD to HD, although, in our cohort, more patients with diabetes mellitus switched to HD (28.2%) compared to nondiabetics (21.3%). This lack of statistical significance could be due to our smaller sample size. Parallel to previous studies, we found no effect of age on transfer rate from PD to HD [17], suggesting that PD can be performed in any age group with appropriate support.

There are little data looking at the association of BMI with technique survival among PD patients. In our study, higher BMI was independently associated with increasing risk of switching from PD to HD. This is in concordance with a recent retrospective cohort study [33] and another study from Australia and New Zealand, in which PD technique failure was 17% higher in obese patients compared to patients with normal BMI [34]. Peritoneal dialysis patients with higher BMI may be at increased risk for not only infectious complications and inadequate dialysis but also peritoneal leaks because of raised intra-abdominal pressure [35,36]. Although in our study there was a clear trend towards a lower risk of transfer from PD to HD for patients living 30 miles or more from their dialysis clinic, this association was not statistically significant. However, a recent report from Canada clearly showed a significant trend toward decreasing PD technique failure with increasing distance from their nephrologist [37].

The impact of dialysis modality switching from PD to HD on patient survival remains controversial. We found no significant difference in survival over time between PD patients who switched to HD compared to those who remained on PD. Similar results have been reported in black patients in the United States [38] and in European cohorts [7]. However, other studies have shown higher mortality for PD patients who switched to HD compared to those who remained on PD [9,19]. In contrast to these reports, Van Biesen et al. [39], found a much better prognosis for PD patients who switched to HD compared to those remaining on PD. These differences in outcomes may be explained by differences in case-mix and reasons for technique failure. Several of these studies, including our own, showed that PD technique failure does not necessarily indicate worse prognosis after switching to HD; rather, more importantly, a timely transfer is vital when severe PD-related complications occur [40].

There are some limitations associated with our study. We had some, but not detailed, data on residual urine output. Furthermore, we had no data on peritoneal membrane characteristics; high peritoneal solute transport has been associated with PD technique failure and mortality in observational studies [23,41] but not in a more recent prospective, randomized, controlled trial [42]. Also, because of the relatively smaller sample size, we combined automated PD and continuous ambulatory PD. But recently, Mujais et al. [24], using data from the Baxter Healthcare Corporation On-Call system reported that transfer to HD was lower in patients on automated PD than in patients on continuous ambulatory PD. However, compared to administrative data, our study provided the advantage of a prospective incident cohort with detailed data on comorbidities, laboratory values, and access to patient charts to determine specific causes of switching. There was a notable rate of lost to follow-up; however, mortality results including passive follow-up were similar to those without passive follow-up. Despite these limitations, our study represents, to our knowledge, one of the few prospective incident cohort studies specifically in the United States analyzing in detail switching of incident PD patients to HD, in terms of rate, timing, predictors and prognosis.

## Conclusion

This prospective study of incident PD patients in the United States confirmed that the observed early switching rate from PD to HD remains too high and certainly represents a significant impediment to the long-term viability of any PD program. Additional efforts are urgently needed to continue to prevent PD-related infections, the leading cause of PD technique failure; also, when antibiotic response for peritonitis remains inadequate, early Tenckhoff catheter removal may help preserve the peritoneal membrane for future return to PD [43]. Moreover, we were able to identify important independent risk factors for switching from PD to HD (BMI and black race). In this context, more studies are definitely needed to better understand why black PD patients were more likely to switch to HD over time. Finally, our findings of no survival difference between PD switchers and non-switchers should be reassuring to providers and patients that PD technique failure is not necessarily associated with poor prognosis, but a timely transfer in setting of complications remains important.

## Competing interests

The authors declare that they have no competing interests.

## Authors' contributions

BGJ analyzed and interpreted the data, reviewed medical records for causes of switching from peritoneal dialysis to hemodialysis, drafted and revised the manuscript for important intellectual content. LCP performed the statistical analysis, analyzed and interpreted the data, and revised the manuscript for important intellectual content. DCC reviewed medical records for causes of switching from peritoneal dialysis to hemodialysis and revised the manuscript for important intellectual content. NEF conceived and designed the study, obtained the data and revised the manuscript for important intellectual content. NH was involved in the provision of study patients and revised the manuscript for important intellectual content. JC conceived and designed the study, obtained the data, obtained funding, supervised the study, and revised the manuscript for important intellectual content. ASK was involved in the provision of study patients and revised the manuscript for important intellectual content. NRP conceived and designed the study, obtained the data, analyzed and interpreted the data, obtained funding, supervised the study, and revised the manuscript for important intellectual content. All authors read and approved the final manuscript.

## Pre-publication history

The pre-publication history for this paper can be accessed here:

http://www.biomedcentral.com/1471-2369/10/3/prepub
